# Dengue virus type 2: replication and tropisms in orally infected *Aedes aegypti *mosquitoes

**DOI:** 10.1186/1471-2180-7-9

**Published:** 2007-01-30

**Authors:** Ma Isabel Salazar, Jason H Richardson, Irma Sánchez-Vargas, Ken E Olson, Barry J Beaty

**Affiliations:** 1 Arthropod-borne and Infectious Diseases Laboratory (AIDL). Department of Microbiology, Immunology and Pathology, Colorado State University, Fort Collins, Colorado. 80523-1692, USA

## Abstract

**Background:**

To be transmitted by its mosquito vector, dengue virus (DENV) must infect midgut epithelial cells, replicate and disseminate into the hemocoel, and finally infect the salivary glands, which is essential for transmission. The extrinsic incubation period (EIP) is very relevant epidemiologically and is the time required from the ingestion of virus until it can be transmitted to the next vertebrate host. The EIP is conditioned by the kinetics and tropisms of virus replication in its vector. Here we document the virogenesis of DENV-2 in newly-colonized *Aedes aegypti *mosquitoes from Chetumal, Mexico in order to understand better the effect of vector-virus interactions on dengue transmission.

**Results:**

After ingestion of DENV-2, midgut infections in Chetumal mosquitoes were characterized by a peak in virus titers between 7 and 10 days post-infection (dpi). The amount of viral antigen and viral titers in the midgut then declined, but viral RNA levels remained stable. The presence of DENV-2 antigen in the trachea was positively correlated with virus dissemination from the midgut. DENV-2 antigen was found in salivary gland tissue in more than a third of mosquitoes at 4 dpi. Unlike in the midgut, the amount of viral antigen (as well as the percent of infected salivary glands) increased with time. DENV-2 antigen also accumulated and increased in neural tissue throughout the EIP. DENV-2 antigen was detected in multiple tissues of the vector, but unlike some other arboviruses, was not detected in muscle.

**Conclusion:**

Our results suggest that the EIP of DENV-2 in its vector may be shorter that the previously reported and that the tracheal system may facilitate DENV-2 dissemination from the midgut. Mosquito organs (e.g. midgut, neural tissue, and salivary glands) differed in their response to DENV-2 infection.

## Background

Dengue is an arthropod-borne viral disease of major public health importance worldwide. Currently, the dengue pandemic affects more than 100 countries, including nations in Africa, the Americas, the Indian subcontinent, Southeast Asia, the Eastern Mediterranean, and the Western Pacific [1]. The World Health Organization (WHO) estimates there are 50 million annual cases of dengue infection worldwide, about 500,000 hospital admissions, and 22,000 dengue-related deaths. Globally, more than 2.5 billion people are at risk for this disease. The emergence of dengue in the Americas has been especially dramatic [2,3].

Dengue virus (DENV) is comprised of four closely related, but antigenically distinct serotypes (DENV-1, DENV-2, DENV-3 and DENV-4), multiple genotypes enclosed in each serotype [4]. The *Aedes aegypti *mosquito is the primary vector for all four serotypes. The mosquitoes become infected after feeding on a viremic individual, then a reported EIP of 7–14 days is required before the mosquitoes can transmit the virus to a new host [2,5]. Environmental factors (including temperature and humidity) as well as intrinsic factors (such as vector competence and viral genotype) affect the EIP [6].

Host organ and cell susceptibility and permissiveness condition virus tropisms in an organism [7]. A number of studies have investigated DENV-vector interactions [8-14], and the important role of virus genotype in efficient *Aedes aegypti *infection has been revealed [15]. In this study, we wanted to determine the virogenesis of DENV-2 infection in recently colonized vector mosquitoes from a dengue endemic area, which previously had been shown to be very susceptible to DENV infection [16].

In the natural course of infection for the virus, DENV is ingested in a blood meal, then it encounters and must overcome midgut infection and escape barriers [17], which vary among mosquito populations. Infection of mosquito midguts is known to occur in a dose-dependent manner [16]. Elucidation of the kinetics of replication and tropism of virus in recently colonized (field relevant) *Aedes aegypti *mosquitoes would provide a better understanding of transmission potential and vector-virus interactions that condition dengue epidemiology and epidemic potential. In this study, we determined: 1) the kinetics of DENV infection in orally infected mosquitoes, 2) the DENV-2 tropisms for different mosquito tissues and organs, 3) a possible anatomic explanation for virus dissemination from the midgut, and 4) the minimal time required for infection of salivary glands in recently colonized *Aedes aegypti *mosquitoes from Chetumal, Mexico and in a long colonized or a selected *Aedes aegypti *strain.

## Results

### *Aedes aegypti *mosquito susceptibility to DENV-2 Jam1409

**Figure 1:**
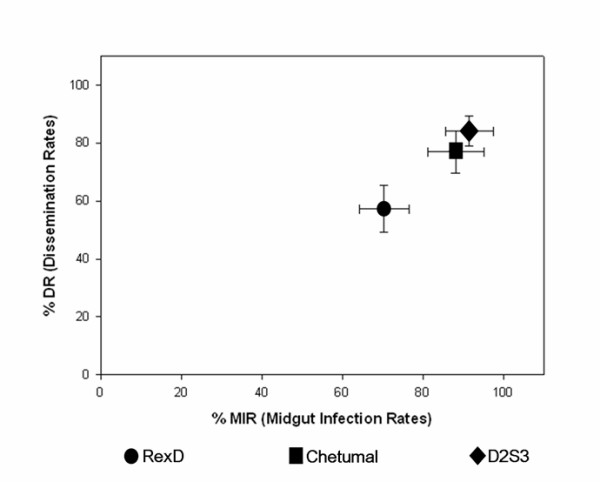
**DENV-2 midgut infection (MIR) and dissemination rates (DR) in *Aedes aegypti *mosquitoes**. MIRs and DRs for Chetumal, Rex-D, and D2S3 mosquito strains at 14 dpi as described in Materials and Methods. The mean and standard deviation values were obtained from four independent experiments (n= 50–60 mosquitoes).

The three *Aedes aegypti *mosquito strains tested were susceptible to DENV-2 Jam1409 midgut infection and dissemination, as determined by detection of DENV-2 antigen in midguts and head tissues at day 14 post blood infection (dpi) (Figure 1). The midgut infection rates (MIR) were similar between Chetumal (87 ± 8%) and D2S3 (90 ± 7%), while they were lower in Rex-D (70 ± 7%) mosquitoes. The dissemination rates (DR) in Chetumal mosquitoes were greater (78 ± 10%) than in the laboratory-adapted Rex-D mosquitoes (57 ± 10%). As expected, the greatest dissemination rates were observed in the D2S3 mosquitoes (90 ± 7%). An ANOVA-one way analysis indicated that means were significantly different for MIR (P = 0.006) as well as for DR (P = 0.002) among the three mosquito populations. The MIR and DR between Chetumal and D2S3 mosquitoes did not differ significantly.

### DENV-2 Jam1409 initial infection, replication, and modulation of infection in midguts

**Figure 2:**
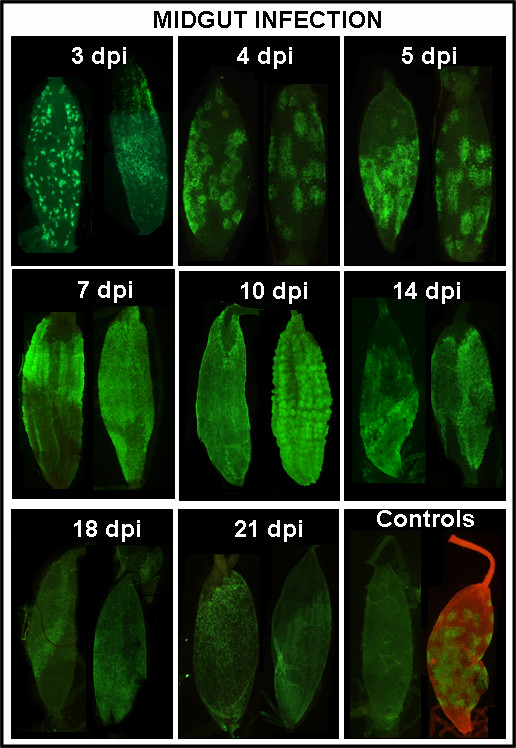
**Kinetics of DENV-2 midgut infection after oral challenge of *Aedes aegypti***. Chetumal mosquito midguts dissected at different time points (n = 30 for each time point) were assayed by IFA to detect DENV-2 viral antigen (green). At each time point two representative midguts from Chetumal mosquitoes are presented. Controls are an uninfected midgut and a dengue infected midgut (in red midgut musculature). The magnification was 100×.

After ingestion of DENV-2, midguts were the first tissues to become infected. At 2 dpi, a few individual infected epithelial cells were distinguishable in ~30% of midguts. By day 3, midgut infection foci involved multiple cells. Infection spread laterally from this initial stage to the neighboring cells (probably due to virus release by budding as observed in invertebrate cells), frequently involving the entire organ by 7–10 dpi. Then, starting at day 10, but more noticeably at 14 dpi, the amount of viral antigen in the midgut began to decline. At 21 dpi viral antigen was almost undetectable, by either the polyclonal or monoclonal anti-dengue antibodies, in midguts of Chetumal (Figure 2) and in Rex-D control mosquitoes (data not shown). In contrast, other organs and tissues, including fat body, salivary glands, and nervous system in the same Chetumal mosquitoes contained large amounts of virus antigen during the time course.

**Figure 3:**
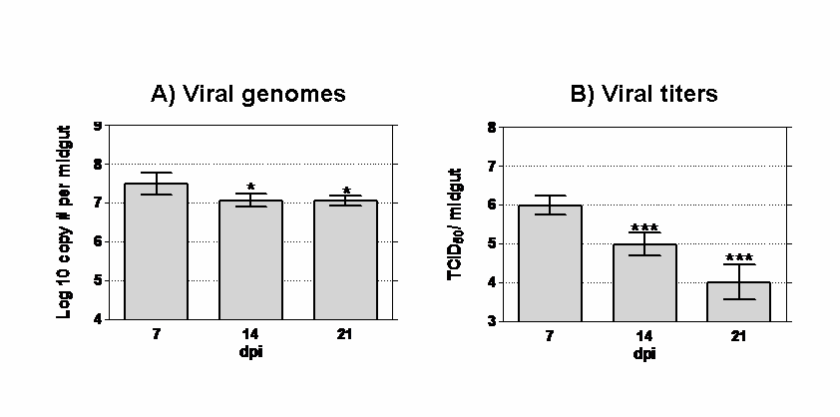
**Quantification of DENV-2 RNA and infectious virus in midguts revealed significant differences**. Midguts (n = 15) from orally infected Chetumal mosquitoes were assayed to quantify viral RNA by Q-PCR and infectious particles by end point titrations at different time points. **A) **Viral RNA decreased slightly between 7 and 14 dpi and then remained stable, * P < 0.05. **B) **Viral titers declined significantly at 14 and 21 dpi, *** P < 0.0001.

DENV-2 + sense RNA as well as infectious virus in Chetumal mosquito midguts was quantified by Q-PCR and end point titration, respectively, at 7, 14 and 21 dpi. In this time frame, the viral RNA remained stable (between 7.2 to 7.6 Log_10 _RNA copy/midgut) as determined by Q-PCR (Figure 3-A). In contrast, between 7 and 21 dpi, virus titers decreased from 6 to 4 Log10 TCID_50_/midgut (P < 0.001) as determined by end point titrations (Figure 3-B).

### Presence of viral antigen in the tracheal system correlates with DENV-2 dissemination in Chetumal mosquitoes

**Figure 4:**
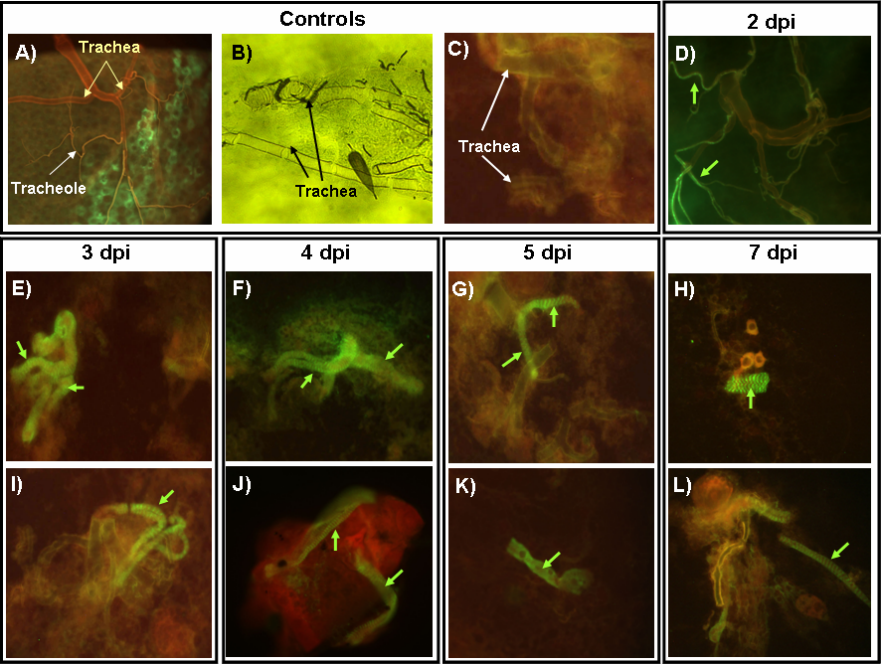
**DENV-2 antigen in the tracheal system of Chetumal mosquitoes following infection**. DENV-2 antigen was detected by IFA in trachea of Chetumal mosquitoes at 2, 3, 4, 5, and 7 dpi (n= 40–50). Midgut- associated tracheal system (A) and uninfected trachea using light (B) and fluorescence (C) microscopy. Green arrows point to portions of DENV-2 infected trachea (E-L) (200×).

Analysis of mosquitoes between 2 and 10 dpi revealed the presence of DENV-2 antigen in portions of the tracheal system in 35 ± 5% of blood fed mosquitoes. The earliest detection of DENV-2 antigen in the tracheal system occurred at 2 dpi, continued at 3, 4, 5, and 7 dpi and then decreased notably. Viral antigen was not detected through out the tracheal system; rather it was confined to small sections of trachea mostly from the abdominal area and it displayed a characteristic distribution pattern (Figure 4). The presence of viral antigen in trachea and virus dissemination out of the midgut in Chetumal mosquitoes were strongly correlated at 2, 3 and 5 dpi (Table 1). This association was gone by 7 dpi (Table 1) and thereafter (data not shown). The criterion for disseminated infection was the presence of viral antigen in any organ or tissue other than the midgut or the tracheal system: early dissemination was detected most frequently in abdominal and throracic fat body, salivary glands and nerve cells and tissues. In contrast to the Chetumal mosquitoes, viral antigen was rarely associated with trachea of infected Rex-D mosquitoes.

### DENV-2 disseminates rapidly to salivary glands in Chetumal mosquitoes

**Figure 5:**
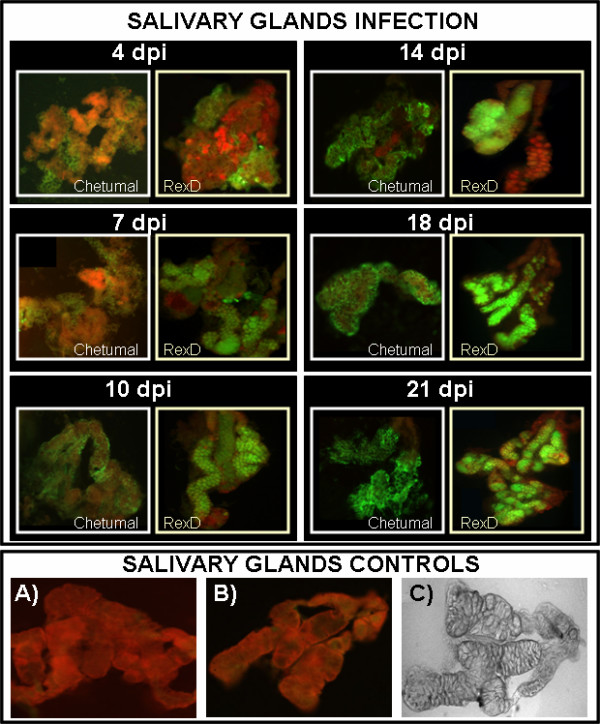
**DENV-2 tropism for *Aedes aegypti *salivary glands**. Salivary glands were dissected at the time points noted, fixed, and assayed for virus antigen by IFA (n = 30–50). Representative salivary glands (200×) from Chetumal and Rex-D mosquitoes at each time point are shown. DENV-2 virus antigen increased in the salivary glands through out the EIP. Negative controls (A and B) and salivary glands examined with light microscopy (C) are shown. In Rex-D mosquitoes, at 4 dpi viral antigen was typically associated with the fat body surrounding the salivary glands. In contrast salivary gland tissues of Chetumal mosquitoes contained viral antigen at 4 dpi.

DENV-2 was detected in the salivary glands of 36% of blood fed Chetumal mosquitoes at 4 dpi. In the Rex-D mosquitoes, this salivary gland infection rate did not occur until 10 dpi and at 7 dpi only < 15% of salivary glands of Rex-D mosquitoes contained viral antigen. Early on the infection, the distal region of the lateral lobes was frequently the area of the salivary gland to contain viral antigen; medial and proximal lobes became infected later. The cells in the distal lateral lobe may contain DENV receptors or more receptors, but this remains to be determined. The percentage of infected salivary glands as well as the amount of viral antigen in the gland increased overtime, revealing a major DENV-2 tropism for this tissue (Figure 5).

### DENV-2 tropisms for various tissues and organs in mosquito

**Figure 6:**
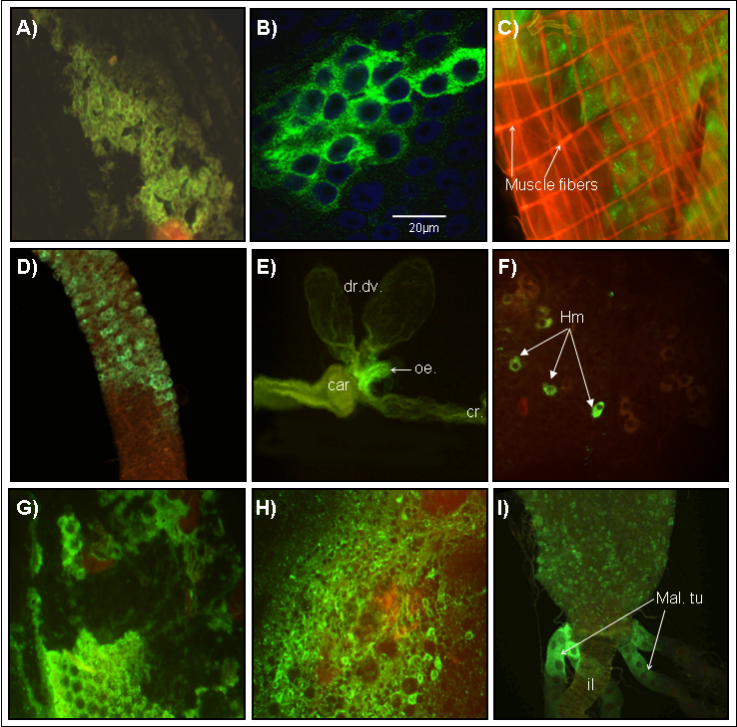
**DENV-2 tropisms in different tissues and organs of *Aedes aegypti***. DENV-2 viral E antigen distribution in different tissues and organs was revealed by IFA (green for FITC or Alexa 488) in Chetumal mosquitoes (n = 20). A) DENV-2 antigen in fat body of mosquito abdomen at 2 dpi. B) DENV-2 infected epithelial cells in midgut (4 dpi). C) DENV-2 is present in epithelial cells but absent in midgut-associated muscle (red phalloidin-Alexa^® ^546). D) DENV-2 in anterior midgut at 5 dpi. E) Infected esophagus at 7 dpi, dr.dv. dorsal diverticulum, car: cardia, oe: esophagus, and cr: crop. F) Hemocytes infected with DENV-2 at 10 dpi. G) Ommatidia of the compound eye exhibiting viral antigen at 12 dpi. H) Nervous system tissue profusely infected by DENV-2 at 14 dpi. I) Malphigian tubules expressing viral antigen (Mal. Tu.) at 16 dpi, il: ileum. These pictures represent the infection patterns observed. Original magnification was 200×, but pictures were cropped in Adobe Photoshop to improve presentation.

The tropisms of DENV-2 Jam1409 to organ-tissues other than midguts and salivary glands were determined over time in Chetumal mosquitoes. DENV-2 antigen was detected in head tissues at 5 dpi, and antigen continued to accumulate in these tissues throughout the EIP. Viral antigen was detected in fat body of the abdominal, thoracic or cephalic regions consistently throughout the time course. DENV-2 dissemination to abdominal fat body was detected in 35 % of mosquitoes between 2 and 3 dpi (Figure 6, panel A) and in 62% of abdominal tissues at the other time points. Thus, fat body represents a major site for DENV-2 replication in the vector. Interestingly the fat body also plays a key role in triggering the insect immune response against pathogens. Hence, it would be important to determine potential effects of virus infection of the fat body on vector competence.

**Figure 7:**
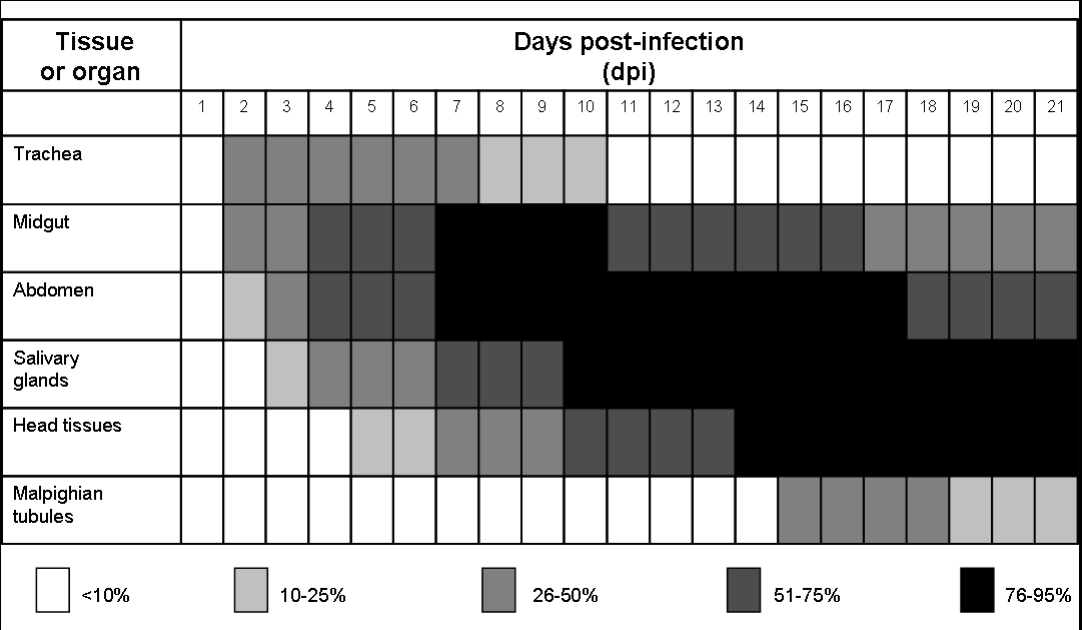
**Temporal tropisms of DENV-2 in Chetumal mosquitoes**. Infection rates in the respective organ or tissue are shown by the gray scale. Percentages were obtained after dissecting, staining and analyzing individual mosquitoes (n = 20–30) at each time point. Mosquitoes were assayed at 1, 2, 3, 5, 7, 10, 14, 18 and 21 dpi.

The musculature surrounding the midgut did not contain virus antigen at any of the examined time points in any of the mosquito populations included in our study (Figure 6-C). Other organs and infected tissues included: trachea, esophagus (Figure 6-E), hemocytes (Figure 6-F), ommatidia of the compound eye (Figure 6-G), nerve tissue (Figure 6-H), and malphigian tubules (Figure 6-I). Occasionally DENV-2 antigen was observed in anterior midgut (Figure 6-D) and not in the hindgut (Figure 6-I) or cardia (Figure 6-E). Taken together, these data revealed the general kinetics of infection and tropisms of DENV-2 in mosquito as shown in Figure 7.

### Kinetics of DENV-2 viral titers in Chetumal mosquitoes

**Figure 8:**
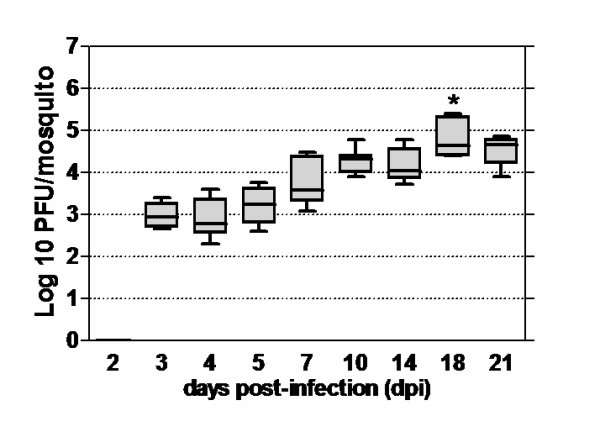
**Kinetics of DENV-2 replication in orally infected mosquitoes**. DENV-2 titers in individual Chetumal mosquitoes were determined by plaque assay at 3, 4, 7, 10, 14, 18 and 21 dpi. Each box in the graph represents mean and range values of viral titers (n = 10) in mosquitoes for each time point.

To establish the overall kinetics of DENV-2 replication, viral titers were determined by plaque assay of individual mosquitoes at different time points (Figure 8). DENV-2 titers increased over time and ranged from 2.2 to 5.5 Log10 PFU/mosquito. Infectious virus was first detectable at 3 dpi. Two slight drops in the overall viral titers were observed: one at 14 dpi and the other at 21 dpi. Kinetics of virus replication during the DENV-2 infection at various time points are shown in Figure 8.

## Discussion

Each of the mosquito strains examined were susceptible to DENV-2. However, the Chetumal and D2S3 strains were clearly more susceptible to DENV-2 infection than the Rex-D mosquitoes (Figure 1). DENV-2 replication in infected Rex-D mosquitoes was also slower than in Chetumal mosquitoes (data not shown). Unfortunately, we did not have a long colonized strain of Chetumal mosquitoes to compare to the newly colonized strain. Colonization has been shown to select for certain genotypes in *Aedes aegypti *[18]. Thus the use of long-colonized mosquitoes as well as high passaged viruses from an area could confound the interpretation of the actual epidemic potential of DENV in that area. It would be prudent to study vector-pathogen interactions using field-relevant mosquitoes as well as low passage viruses from a specific area in order to draw more precise conclusions about the risk of dengue transmission in that area.

It was surprising that the Chetumal mosquitoes were as permissive to DENV-2 productive infection as the D2S3 strain, which was selected for DENV susceptibility [19]. The susceptible phenotype is likely conditioned by different genetic mechanisms, which remain to be determined. However, the results do confirm previous studies demonstrating that mosquitoes from the eastern portion of the Yucatan Peninsula (an endemic area for dengue) are remarkably competent vectors for DENV [16].

DENV-2 infection was detected in midgut epithelial cells as early as 2 dpi. Viral titers and virus antigen then increased until 10 dpi when both virus titer and antigen decreased (Figure 2 and 3). In contrast, Q-PCR revealed that viral RNA copy number remained fairly constant even late into the EIP (Figure 3). Conversely, DENV-2 antigen was not cleared from salivary glands or neural tissues at equivalent time points. The former observation is of interest, because once a mosquito is productively infected with an arbovirus, she is capable of transmitting the virus for life. Obviously, any virus modulation in the salivary glands would be epidemiologically significant since transmission efficiency could be reduced. Modulation of virus in the midgut is provocative; either the virus or the epithelium is active in modulating the virus infection. The continued presence of DENV-2 RNA suggests a probable mechanism of persistence in vectors. DENV-2 as other arboviruses can persistently infect insect and human cell lines and arthropod vectors [20-23]. It is unlikely that the modulation is conditioned by the metabolic activity of the midgut epithelium, because a second uninfectious bloodmeal provided 14 days after DENV-2 infection of *Aedes aegypti *failed to reestablish virus production in the midgut (data not shown). The decline in infectious virus and viral antigen may be associated with an antiviral response, post-transcriptional or post-translational repression, or other self-limiting factors such as epithelial cells physiological status (i.e. cell aging). Further studies will be necessary to elucidate the molecular determinants of this phenomenon.

The mechanism(s) by which DENV disseminates from the midgut are not well understood. This study suggests that the mosquito tracheal system may be implicated in DENV dissemination (Figure 4 and Table 1). The presence of virus antigen in tracheal system significantly correlated with DENV-2 dissemination, mostly between 3 and 5 dpi. Tracheoles are part of the tracheal system, are 0.2 to 1 μm in diameter, are fluid-filled, and reach every tissue in the insect. The tracheal system has been reported to act as dissemination conduit for different viruses in insects. Notable examples are *Autographa californica *M nuclear polyhedrosis virus (AcMNPV) infecting *Trichoplusia ni*, nucleopolyhedrovirus infecting *Bombyx mori*, and Sindbis virus in *Aedes albopictus *[24,25]. The tracheal system is also suspected to be the midgut escape conduit for Venezuelan equine encephalitis virus (VEEV) in *Ochlerotatus taeniorhynchus *[26]. However, this is the first report indicating that DENV may use the mosquito tracheal system during dissemination.

Unlike a number of other arboviruses, DENV displayed no tropism for midgut-associated (Figure 6-C) or flight muscles. Rift Valley Fever virus infects both skeletal and visceral muscles [27]. Sindbis virus replicates in visceral muscles of *Aedes albopictus *[28]. West Nile virus infects the contiguous muscles of the posterior and anterior midgut in *Culex *mosquitoes [29]. The reasons for mosquito muscle refractoriness to DENV are unknown and require further analysis. Rift Valley fever virus and Sindbis alphavirus exhibit tropisms for cardia tissue [11,30], while DENV-2 was not routinely found in this tissue.

Interestingly, DENV-2 infection was detected in 36% of Chetumal mosquito salivary glands by 4 dpi. In contrast, < 15% of Rex-D mosquitoes had infected salivary glands at 7 dpi and salivary gland infection rates >36% weren't detected until 10 dpi. The Chetumal mosquitoes would seem to be more vector competent than the Rex-D mosquitoes. Reportedly the EIP for DENV is between 7 and 14 days, and it can be affected by conditions such as temperature, humidity and viral dose [2,5,6,31]. Blood meals provided to mosquitoes in our studies contained 1.7 ± 0.7 × 10^7 ^PFU/ml, which is likely biologically relevant, since viremia titers of DENV-2 in humans may vary from 10^2 ^to 10^7 ^PFU/ml or 10^3 ^to 10^8 ^TCID_50_/ml [32,33]. In our observations, the lateral distal lobes were frequently the first site to become infected in the salivary glands, the medial and proximal lobes developed infection later (Figure 5). This pattern probably results from the higher virus receptor concentration in this region of the salivary glands, but this remain to be determined. The lateral distal lobes participate prominently in the secretion of enzymes and proteins involved in hematophagy [34,35], therefore infection of this region might promote early virus transmission. Other studies have demonstrated that DENV-2 can be transmitted by mosquitoes with < 25% salivary gland tissue infected, if infection occurred in lateral lobes [31]. Thus, our results suggest that the EIP for DENVs may be shorter than the commonly reported (at least with some strains of the vector). There is no sensitive and reliable laboratory animal model for dengue, and currently used *in vitro *assays for transmission may fail to detect small but transmissible amounts of virus at earlier times. However further studies will be necessary to demonstrate that infectious virus is being transmitted early in the EIP and that the results can be extrapolated to low passaged DENV strains.

A shorter EIP for dengue would have important epidemiological consequences in dengue transmission. In nature, *Aedes aegypti *female mosquitoes feed frequently and almost exclusively on humans, which confers greater fitness than feeding on other hosts [36]. These highly anthropophilic mosquitoes may ingest 2 to 3 bloodmeals during a single gonadotropic cycle, feeding with a frequency of 0.63 to 0.76 times a day [37]. *Aedes aegypti *mosquitoes, with a very short EIP, persistently infected salivary glands, and the ability to feed multiple times within a gonadotropic cycle, would seem to be the ultimate DENV vector, which could help explain the epidemic potential of dengue.

## Conclusion

The results with the recently colonized Chetumal mosquitoes indicate that the EIP for DENVs may be shorter than the commonly reported, which could have important epidemiological consequences in dengue transmission. The mosquito tracheal system appears implicated in virus dissemination from the midgut, and viral infection in the vector is differentially modulated in different organs. DENV-2 exhibits differences in certain tissue tropisms in its vector in comparison to other arboviruses in their respective vectors.

## Methods

### Mosquito rearing

The newly-colonized *Aedes aegypti *Chetumal strain was characterized for its vector competence for DENV-2 [16,38]. Chetumal mosquitoes (F3 – F6) were originally collected from in the Yucatan Peninsula, Mexico. Mosquitoes were hatched and reared as reported previously [16,17,38]. After hatching female pupae were separated from males by size, since males are significantly smaller; transferred in plastic cups to cardboard cartons and allowed to emerge. Adult mosquitoes were maintained at 28°C, 70–80% relative humidity, with a 12–12 h light-dark photoperiod. Mosquitoes used as controls in tropism experiments were the Puerto Rico-Rex-D strain (a long colonized laboratory strain) [39] and the D2S3 strain (a laboratory selected, highly susceptible mosquito strain lacking a midgut escape barrier) [19].

### Virus propagation

A high passage DENV-2 strain (Jamaica 1409) [40] was obtained from the Centers for Diseases Control and Prevention (CDC- Fort Collins, CO, USA). The virus had been routinely passaged (> 25 times) in C6/36 cell culture, but there were only two relevant changes in comparison to the low passaged virus as the full-length sequence analysis indicated [41]. These changes were a conservative amino acid substitution in E_6 _protein (Ile→Met) and a nucleotide change in the 3'UTR_143 _(G→T). The C6/36 cell cultures were infected at a multiplicity of infection (MOI) of 0.001 and incubated at 28°C for a week. At day 7, the medium was replaced with fresh medium, and at 12 days post-infection virus was harvested to prepare infectious bloodmeals [16,42].

### Oral infection of mosquitoes with DENV-2

*Aedes aegypti *mosquitoes were challenged with DENV-2 Jam1409 using an artificial bloodmeal [16]. Briefly, 100–150 *Aedes aegypti *female mosquitoes, 4 – 7 days post-emergence, were deprived of sugar for 24 h and water for 8 h prior to the bloodfeeding. The virus-infected cell monolayers were scraped from the flask surface, and 2 ml of this suspension was added to an equal amount of washed sheep erythrocytes to prepare the infectious bloodmeal, which was provided to the mosquitoes using a membrane feeding device. The meal was maintained at 37°C by circulating warm water though an external jacket. Mosquitoes were not allowed to feed longer than 45 min to prevent a decline in viral titers. Blood meals contained viral titers of 1.7 ± 0.7 × 10^7 ^PFU/ml. Blood fed mosquitoes were separated using a chill table, transferred to clean cartons, and maintained at as noted above.

### Indirect immunofluorescence assays (IFA)

To determine viral tropisms, mosquito (n = 30–40) tissues were examined for the presence of virus antigen after dissecting midguts and salivary glands and squashing their thoraxes, heads, and abdomens on slides.

Midguts were dissected in PBS and fixed in 4% paraformaldehyde (Electron Microscopy, Hatfield, PA) for at least 4 h. The fixative was then removed, rinsed once in PBS, and then incubated for 1 h at room temperature with constant rocking in a solution containing: 1× PBS-Ashburner's solution (13 mM NaCl, 0.7 mM Na_2_HPO_4_, 1 mM NaH_2_PO_4 _at pH = 7.2), 1% Bovine Serum Albumin, and 0.1% Triton X-100, called PBT-0.1 (PBS-BSA-Triton). Typically to detect DENV-2 antigen by IFA, the primary antibody was the serotype-specific mouse 3H5–1 monoclonal antibody (ATCC number HB-46). To confirm the decline in DENV-2 antigen in midguts, a polyclonal human anti-dengue specific antibody (Biodesign International, Saco, ME) was also used as the primary antibody. Midguts were then incubated with a 1:400 dilution of goat-antimouse antibody conjugated with Alexa-488 fluorochrome (Molecular Probes Inc, Eugene, OR). Midguts were washed twice with PBT-0.1 with constant rocking at room temperature for 2 hr and incubated with a 1:40 dilution of Phalloidin-Alexa 546 (Molecular Probes Inc, Eugene, OR) for 20 min. Midguts were rinsed with PBS and placed onto slides, covered with Vectashield^® ^(Vector Laboratories Inc, Burlingame, CA), and a coverslip was sealed with nail polish on the slide. Preparations were examined using the Olympus BH2-RFCA epifluorescence microscope or a confocal microscope (FVX-IHRT Fluoview Confocal, LSM, Olympus).

Salivary glands were dissected in PBS onto Vectabond^® ^(Vector Laboratories Inc, Burlingame, CA) pretreated slides, fixed in cold acetone or fixed in 4% paraformaldehyde- 2% glutaraldehyde, and maintained at 4°C until immunosassay. Abdomens, thoraxes and heads were squashed on glass Vectabond^®^-pretreated slides, fixed in cold acetone for 15 min, and kept at 4°C

IFA was used to detect DENV-2 antigen in salivary glands, thoraxes, abdomens and heads. The primary serotype-specific monoclonal DENV-2 antibody 3H5-1 was diluted 1:200 in PBS and incubated for 1 h on the tissue. Slides were then washed twice for 5 min with PBS, and goat-anti-mouse avidin-conjugated secondary antibody containing 0.01% Evans Blue (1:200; Amersham Pharmacia Biotech, Piscataway, NJ) was added. After 40 min, samples were washed 2× in PBS and incubated for 20 min with a 1:200 dilution of fluorescein-streptavidin (Amersham Pharmacia Biotech, Piscataway, NJ). Following two additional PBS washes of 5 min each and one with distilled water, DABCO oil was added to the tissues and coverslips were added. All the incubations were performed at 37°C. An Olympus BH2-RFCA fluorescence microscope was used to examine slides.

### Determination of midgut infection and dissemination rates

The midgut infection rate (MIR) was determined as the number of midguts containing DENV-2 antigen divided by the number of midguts examined. The dissemination rate (DR) was determined as the number of mosquitoes with detectable DENV-2 antigen in non-midgut tissues (eg, head squash tissues, salivary glands, fat body, etc.), divided by the number of mosquitoes with detectable virus antigen in the midgut.

### Virus titration

Viral titers in midguts were determined by end point titrations (TCID_50_) in C6/36 cells. Titers were established using the microtiter plate titration assay described by Schoepp and Beaty [43], and endpoints were calculated by the Karber method [44] and expressed as log_10 _TCID_50_/ml. To determine viral titers in whole mosquitoes as well as in some midguts, plaque assay titrations in LLC-MK2 cells were used modifying the protocol originally reported by Malewicz and Jenkin [45].

### DENV- 2 quantitative real time RT-PCR (Q-PCR)

DENV-2 genome copy number was determined Q-PCR in homogenized and filtered tissue from individual mosquitoes or midguts [38][46]. RNA for PCR assays was extracted using QIAamp^® ^Viral RNA Kit (Qiagen Sciences, Maryland, MA). The reaction mixture was prepared with 1× DyNAzyme^® ^buffer (Finnzymes, Espoo, Finland), 0.2 mM dNTP mix, 0.5× SYBRGreen I (Molecular Probes Inc, Eugene, OR), 2.5 mM MgCl_2_, 10 μM of forward and reverse primers (5'CAAGTCGAACAACCTGGTCCAT3' and 5'GCCGCACCATTGGTCTTCTC3' respectively), 0.4U DyNAzyme II Recombinant DNA Polymerase (Finzymmes, Espoo, Finland), and 2 μl cDNA in a final volume of 20 μL. The SYBR Green I 10,000× stock was diluted in DMSO to a working solution of 100×, which was stored at -20°C in 15 μL aliquots to minimize exposure to light and freeze-thaw cycles. Reactions were performed in a microseal 96 microplates covered with optically clear caps (MJ Research, Waltham, MA) and placed in a Opticon 2 thermal cycler. Settings were: 95°C for 10 min, followed by 40 cycles of 95°C for 10 sec, 64°C for 20 sec, 72°C for 30 sec, and 84°C for 1 sec for fluorescence measurement. After a final extension at 72°C for 10 min, a melting curve was ascertained using the program: 70°C to 95°C, 0.2°C/read, 1 sec hold to confirm product specificity.

The primers used targeted a 177 bp region of the DENV-2 NS5 gene and quantified (+) strand DENV-2 RNA in the sample. Standard curves were generated on each plate by analyzing 2 × 10^2 ^to 2 × 10^8 ^copies/reaction of DENV-2 from plasmid standards.

## Authors' contributions

MIS conducted most of the laboratory experiments, analyzed the data and wrote the manuscript. JHR participated in the Q-PCR experiments and data analysis. ISV contributed in the salivary gland studies and in control experiments with Rex-D mosquitoes. KEO supervised the research progress and edited the manuscript. BJB established the international collaboration, coordinated the project, and edited the manuscript. All authors read and approved the final manuscript.

## Figures and Tables

**Table 1:** The presence of DENV-2 antigen in the tracheal system correlates with early dissemination in Chetumal mosquitoes

	**2dpi**		**3dpi**		**5dpi**		**7dpi**	
**DENV-2 infection**	**Trachea**		**Trachea**		**Trachea**		**Trachea**	

	(+)	(-)	(+)	(-)	(+)	(-)	(+)	(-)
Disseminated	5	1	10	4	16	2	5	12
Non-Disseminated	1	12	2	8	1	3	2	3
Total	6	13	12	12	17	5	7	15
χ^2^	10.87		6.17		7.28		0.199	
Significance	ρ < 0.001		ρ < 0.01		ρ < 0.01		ρ >0.05	

The association of detection of viral antigen in the tracheal system with early dissemination of the virus was analyzed using 2 × 2 tables for each dpi. The criterion for dissemination of infection was detection of viral antigen in non-midgut tissues, including head squash tissues, fat body, and salivary glands.

## References

[B1] Derouich M, Boutayeb A, Twizell EH (2003). A model of dengue fever. Biomed Eng Online.

[B2] Gubler DJ (1998). Dengue and dengue hemorrhagic fever. Clin Microbiol Rev.

[B3] Gubler DJ, Clark AG (1995). Dengue/dengue hemorrhagic fever: The emergence of a global health problem. Emerg Infect Dis.

[B4] Holmes EC, Twiddy SS (2003). The origin, emergence and evolutionary genetics of dengue virus. Infect Genet Evol.

[B5] Watts DM, Burke DS, Harrison BA, Whitmire RE, Nisalak A (1987). Effect of temperature on the vector efficiency of Aedes aegypti for dengue 2 virus. Am J Trop Med Hyg.

[B6] Black WCIV, Bennett KE, Gorrochotegui-Escalante N, Barillas-Mury C, Fernandez-Salas I, Munoz ML, Farfan JA, Olson KE, Beaty BJ (2002). Flavivirus susceptibility in Aedes aegypti. Arch Med Rev.

[B7] Flint SJ, Enquist LW, Krug RM, Racaniello VR, Skalka AM (2000). " Molecular Biology, Pathogenesis and Control". Principles of Virology.

[B8] Schoepp RJ, Beaty BJ, Eckels KH (1990). Dengue 3 virus infection of Aedes albopictus and Aedes aegypti: comparison of parent and and progeny candidate vaccine viruses. Am J Trop Med Hyg.

[B9] Schoepp RJ, Beaty BJ, Eckels KH (1991). Infection of Aedes albopictus and Aedes aegypti with dengue parent and progeny candidate vaccine viruses: a possible marker of human attenuation. Am J Trop Med Hyg.

[B10] Vazeille-Falcoz M, Rosen L, Mousson L, Failloux AB (2003). Low oral receptivity for dengue type 2 viruses of Aedes albopictus from Southeast Asia compared with that of Aedes aegypti. Am J Trop Med Hyg.

[B11] Linthicum KJ, Platt K, Myint KS, Lerdthusnee K, Innis BL, Vaughn DW (1996). Dengue 3 distribution in the mosquito Aedes aegypti: an immunocytochemical study. Med Vet Entomol.

[B12] Kuberski T (1979). Fluorescent antibody studies on the development of dengue-2 virus in Aedes albopictus (Diptera:Culicidae). J Med Entomol.

[B13] Miller BR, Ballinger ME (1988). Aedes albopictus mosquitoes introduction into Brazil: vector competence for yellow fever and dengue viruses.. Trans R Soc Trop Med Hyg.

[B14] Vazeille-Falcoz M, Rosen L, Mousson L, Rodhain F (1999). Replication of dengue type 2 virus in Culex quinquefasciatus (Diptera: Culicidae). Am J Trop Med Hyg.

[B15] Armstrong PM, Rico-Hesse R (2001). Differential susceptibility of Aedes aegypti to infection by the American and Southeast Asian genotypes of dengue type 2 virus. Vector Borne Zoonotic Dis.

[B16] Bennett KE, Olson KE, Munoz ML, Fernandez-Salas I, Farfan JA, Higgs S, Black WC, Beaty BJ (2002). Variation in vector competence for dengue 2 virus among 24 collections of Aedes aegypti from Mexico and United States. Am J Trop Med Hyg.

[B17] Bosio CF, Fulton RE, Salasek ML, Beaty BJ, Black WC (2000). Quantitative trait loci that control vector competence for dengue-2 virus in the mosquito Aedes aegypti. Genetics.

[B18] Lorenz L, Beaty BJ, Aitken TH, Wallis GP, Tabachnick WJ (1984). The effect of colonization upon Aedes aegypti susceptibility to oral infection with yellow fever virus. Am J Trop Med Hyg.

[B19] Bennett KE, Beaty BJ, Black WC (2005). Selection of D2S3, an Aedes aegypti (Diptera: Culicidae) strain with high oral susceptibility to Dengue 2 virus and D2MEB, a strain with a midgut barrier to Dengue 2 escape. J Med Entomol.

[B20] Kurane I, Kontny U, Janus J, Ennis FA (1990). Dengue-2 virus infection of human mononuclear cell lines and establishment of persistent infections. Arch Virol.

[B21] Newton SE, Short NJ, Dalgarno L (1981). Bunyamwera virus replication in cultured Aedes albopictus (mosquito) cells: establishment of a persistent viral infection.. J Virol.

[B22] Kuno G (1982). Persistent infection of a nonvector mosquito cell line (TRA-171) with dengue viruses. Intervirology.

[B23] Elliott RM, Wilkie ML (1986). Persistent infection of Aedes albopictus C6/36 cells by Bunyamwera virus. Virology.

[B24] Engelhard EK, Kam-Morgan LNK, Washburn JO, Volkman LE (1994). The insect tracheal system: A conduic for systemic spread of Autographa californica M nuclear polyhedrosis virus. Proc Natl Acad Sci U S A.

[B25] Bowers DF, Abell AA, Brown DT (1995). Replication and tissue tropism of the alphavirus sindbis in the mosquito Aedes albopictus. Virology.

[B26] Romoser WS, Wasieloski LP, Pushko P, Kondig JP, Lerdthusnee K, Neira M, Ludwig GV (2004). Evidence for arbovirus dissemination conduits from the mosquito (Diptera: Culicidae) midgut. J Med Entomol.

[B27] Romoser WS, Faran ME, Bailey CI, Lerdthusnee K (1992). An immunocytochemical study of the distribution of Rift Valley virus in the mosquito Culex pipiens. Am J Trop Med Hyg.

[B28] Bowers DF, Coleman CG, Brown DT (2003). Sindbis virus-associated pathology in Aedes albopictus (Diptera: Culicidae).. J Med Entomol.

[B29] Girard M, Klingler KA, Higgs S (2004). West Nile virus dissemination and tissue tropism in orally infected Culex pipiens. Vector-borne and Zoonotic Dis.

[B30] Lerdthusnee K, Romoser WS, Faran ME, Dohm DJ (1995). Rift Valley fever virus in the cardia of Culex pipiens: an immunocytochemical and ultrastructural study. Am J Trop Med Hyg.

[B31] Gubler DJ, Rosen L (1976). A simple technique for demonstrating transmission of dengue virus by mosquitoes without the use of vertebrate hosts. Am J Trop Med Hyg.

[B32] Shu PY, Chang SF, Kuo YC, Yueh YY, Chien LJ, Sue CL, Lin TH, J.H. H (2003). Development of group- and serotype-specific one-step SYBR green I-based real-time reverse transcription-PCR assay for dengue virus. J Clin Microbiol.

[B33] Craig S, Thu HM, Lowry K, Wang XF, Holmes EC, Aaskov J (2003). Diverse dengue type 2 virus populations contain recombinant and both parental viruses in a single mosquito host. J Virol.

[B34] Smartt CT, Kim AP, Grossman GL, James AA (1995). The Apyrase gene of the vector mosquito, Aedes aegypti, is expressed specifically in the adult female salivary glands. Exp Parasitol.

[B35] Argentine JA, James AA (1995). Characterization of a salivary gland-specific esterase in the vector mosquito, Aedes aegypti. Insect Biochem Mol Biol.

[B36] Harrington LC, Edman JD, Scott TW (2001). Why do female Aedes aegypti (Diptera: Culicidae) feed preferentially and frequently on human blood?. J Med Entomol.

[B37] Scott TW, Amerasinghe PH, Morrison AC, Lorenz LH, Clark GG, Strickman D, Kittayapong P, Edman JD (2000). Longitudinal studies of Aedes aegypti (Diptera: Culicidae) in Thailand and Puerto Rico: blood feeding frequencv.. J Med Entomol.

[B38] Richardson J, Molina-Cruz A, Salazar MI, Black WC (2006). Quantitative analysis of dengue-2 virus RNA during the extrinsic incubation period in individual Aedes aegypti. Am J Trop Med Hyg.

[B39] Gubler DJ, Novak RJ, Vergne E, Colon NA, Velez M, Fowler J (1985). Aedes (Gymnometopa) mediovittatus (Diptera: Culicidae), a potential maintenance vector of dengue viruses in Puerto Rico. J Med Entomol.

[B40] Deubel V, Kinney RM, Trent DW (1986). Nucleotide sequence and deduced aminoacid sequence of nonstructural proteins of dengue type 2, Jamaica genotype: comparative analysis of full-length genome.. Virology.

[B41] Pierro DJ, Salazar MI, Beaty BJ, Olson KE (2005). Infectious clone construction of dengue virus type 2, strain Jamaica 1409 and characterization of a conditional mutation in the envelope glycoprotein. J Gen Virol.

[B42] Bosio CF, Beaty BJ, Black WC (1998). Quantitative genetics of vector competence for dengue-2 virus in Aedes aegypti.. Am J Trop Med Hyg.

[B43] Schoepp RJ, Beaty BJ (1984). Titration of dengue viruses by immunofluorescence in microtiter plates. J Clin Microbiol.

[B44] Karber G (1931). Beitrag zur kollektiven Behandlung pharmakologischer 
Reihenversuche. Arch Exp Pathol Parmakol.

[B45] Malewicz B, Jenkin HM (1979). Development of dengue virus plaques under serum-free overlay medium. J Clin Microbiol.

[B46] Molina-Cruz A, Gupta L, Richardson J, Bennett K, Black WC, Barillas-Mury C (2005). Effect of mosquito midgut trypsin activity on dengue-2 virus infection and dissemination in Aedes aegypti. Am J Trop Med Hyg.

